# The ENCODE Portal as an Epigenomics Resource

**DOI:** 10.1002/cpbi.89

**Published:** 2019-11-21

**Authors:** Jennifer Jou, Idan Gabdank, Yunhai Luo, Khine Lin, Paul Sud, Zachary Myers, Jason A. Hilton, Meenakshi S. Kagda, Bonita Lam, Emma O'Neill, Philip Adenekan, Keenan Graham, Ulugbek K. Baymuradov, Stuart R. Miyasato, J. Seth Strattan, Otto Jolanki, Jin‐Wook Lee, Casey Litton, Forrest Y. Tanaka, Benjamin C. Hitz, J. Michael Cherry

**Affiliations:** ^1^ Department of Genetics Stanford University Stanford California

**Keywords:** database, ENCODE, epigenetics, human genome, regulatory elements

## Abstract

The Encyclopedia of DNA Elements (ENCODE) web portal hosts genomic data generated by the ENCODE Consortium, Genomics of Gene Regulation, The NIH Roadmap Epigenomics Consortium, and the modENCODE and modERN projects. The goal of the ENCODE project is to build a comprehensive map of the functional elements of the human and mouse genomes. Currently, the portal database stores over 500 TB of raw and processed data from over 15,000 experiments spanning assays that measure gene expression, DNA accessibility, DNA and RNA binding, DNA methylation, and 3D chromatin structure across numerous cell lines, tissue types, and differentiation states with selected genetic and molecular perturbations. The ENCODE portal provides unrestricted access to the aforementioned data and relevant metadata as a service to the scientific community. The metadata model captures the details of the experiments, raw and processed data files, and processing pipelines in human and machine‐readable form and enables the user to search for specific data either using a web browser or programmatically via REST API. Furthermore, ENCODE data can be freely visualized or downloaded for additional analyses. © 2019 The Authors.

**Basic Protocol**: Query the portal

**Support Protocol 1**: Batch downloading

**Support Protocol 2**: Using the cart to download files

**Support Protocol 3**: Visualize data

**Alternate Protocol**: Query building and programmatic access

## INTRODUCTION

The Encyclopedia of DNA Elements (ENCODE; https://www.encodeproject.org/) web portal hosts genomic data generated by the ENCODE Consortium, the Genomics of Gene Regulation project, the NIH Roadmap Epigenomics Consortium (Bujold et al., 2016; Kundaje et al., 2015), and the Model organism ENCODE (modENCODE) and modERN projects (Gerstein et al., 2010; Roy et al., 2010; Stamatoyannopoulos et al., 2012). The ENCODE project has the goal of identifying all functional elements in the human and mouse genomes. The ENCODE portal (Davis et al., 2018; Hitz et al., 2017; Hong et al., 2016; Sloan et al., 2016) serves as the canonical source of ENCODE data, and is actively maintained by the ENCODE Data Coordination Center (DCC) to update the relevant experimental data and metadata and provide visualization and analysis tools for the scientific community. For this reason, researchers seeking to use ENCODE data should always use the ENCODE portal to ensure that they get the most up‐to‐date analysis results for their experiments, as well as metadata about data provenance and experimental methods. However, to facilitate data accessibility and findability, ENCODE data are also deposited to other repositories such as GEO (Barrett et al., 2013) and EpiRR (https://www.ebi.ac.uk/vg/epirr).

The protocols covered in this article will allow the scientific community to rapidly find and utilize the open‐access data resources provided by the ENCODE Consortium. The ENCODE portal provides various methods allowing users to search for and download data using a web browser. The Basic Protocol demonstrates how to navigate the ENCODE portal website to retrieve experiment records relevant to one's interests, as well as interpret the presented metadata.

Three support protocols describe additional features available on the portal: Support Protocol 1 describes how to download multiple files from a search result page containing one or more datasets; Support Protocol 2 shows how to save any experiment to a cart, from which files can be downloaded; and lastly Support Protocol 3 reviews methods for rapid visualization of signal and peaks tracks using the embedded Valis genome browser (https://valis.bio/) directly on the portal website or using external resources such as the UCSC Genome Browser (Kent et al., 2002).

The Alternate Protocol presents a method, independent of the portal website, producing the same search results as the Basic Protocol by directly interacting with the REST API, allowing programmatic access to the database.

## QUERY THE PORTAL

Both the Matrix and the Search pages provide an intuitive method to perform metadata‐based searches for ENCODE datasets in the form of facet filters, which are categorized lists of commonly used experimental metadata.

### Necessary Resources

#### Hardware

Computer with internet access

#### Software

Up‐to‐date web browser (Chrome, Microsoft Edge, Firefox, Safari)

### Use the matrix to navigate to a search page

**Figure 1:**
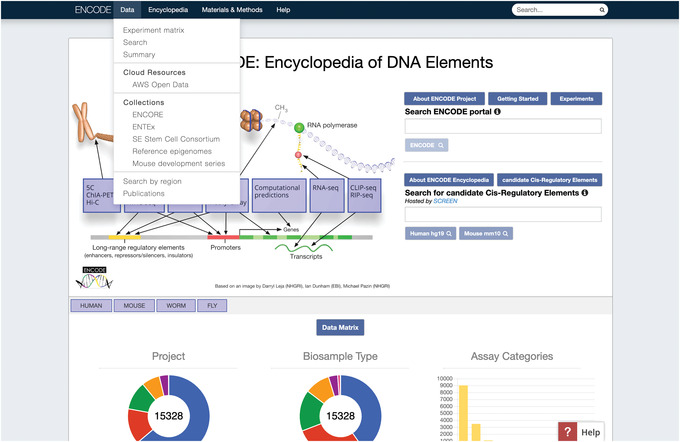
The ENCODE home page. This image shows the Data drop‐down menu in the toolbar opened. The first item in the menu is a link to the Experiment Matrix page.

1 Navigate to the ENCODE portal home page at https://www.encodeproject.org. A clickable widget labeled “Help” automatically loads in the lower right corner of the browser window, which contains links to interactive tutorials, frequently asked questions, and other documentation on topics not addressed in this article. Users are encouraged to explore the widget for additional help with the portal.
2 In the toolbar along the top of the page, click “Data.” This opens a drop‐down menu with multiple options (Fig. 1).
  The menu options in the toolbar provide access to key resources on the portal, including the Experiment matrix page and a link back to the home page. For example, the Data drop‐down menu also has links to Search and Summary pages, which represent the same data as the Experiment matrix page but in different layouts. The Encyclopedia menu contains information about and links to integrative‐level annotations generated using ENCODE data. The Materials & Methods menu links to information about experimental components used, data‐processing methods, and portal‐organization methodology. The Help menu contains links to information about ENCODE, portal usage, past ENCODE workshops, and contact information. A cart menu will also appear here if there are experiments added to the cart, which is discussed in Support Protocol 2.

**Figure 2:**
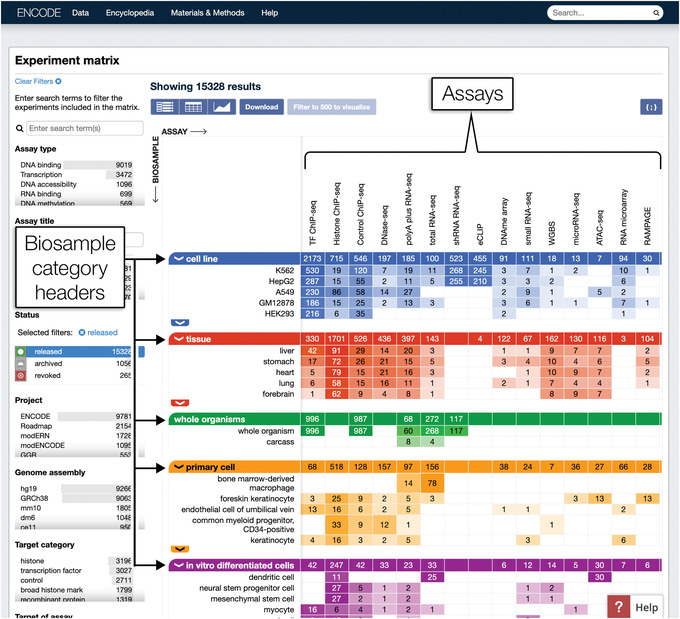
The Experiment Matrix page displays available ENCODE data in a matrix with biosample types and assays as the axes. Each cell of the matrix is clickable and leads to a list of experiments matching the given combination of biosample type and assay.

3 In the drop‐down menu, click “Experiment Matrix” to navigate to the Experiment matrix page (Fig. 2).
  - The Experiment matrix page lists biosample types, which refer to the biological material used such as a cell culture or tissue sample along the *y* axis, and various assays along the *x* axis, with each cell indicating the number of experiments of that combination of assay and biosample type.
  - Only a subsection of the matrix is visible upon loading the page. To view more, click the arrows along the left side of the biosample category headers to expand the categories and reveal an extended list of available biosample types. With the mouse cursor hovering on top of the matrix, scroll horizontally to view more available assays.

**Figure 3:**
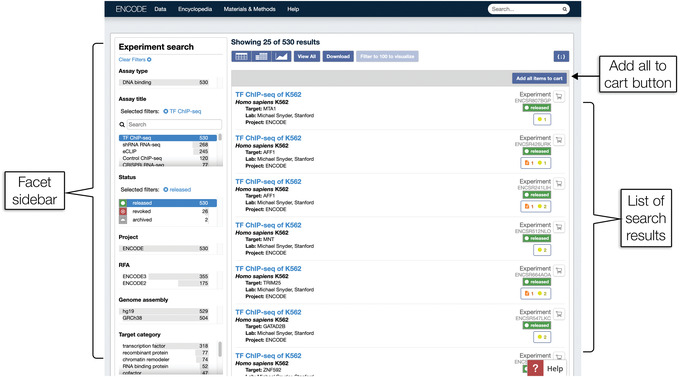
The Experiment search page displays ENCODE data as a list of search results. Each experiment is shown with a brief summary of the biological material and assay name, and a link to its individual experiment summary page with more metadata details (see Fig. 5). On the left is the facet sidebar, which can be used to modify and refine the search results. The “Add all items to cart” button is a Cart function, explained further in Support Protocol 3.

4 Click on the cell for transcription factor ChIP‐seq (abbreviated as TF ChIP‐seq) experiments on K562. As of September 2019, the ENCODE portal had 530 experiments in this group. This link leads to a search page with a list of the 530 experiments, shown in Figure 3.
  By default, only experiments with the status “released” are included in a search. Explanations of the meaning of different experimental statuses are available in Table 1.

**Table 1:** Dataset Statuses^a^

Status	Meaning
Released	Publicly available datasets are marked with the “released” status. Datasets become publicly available after automatic and manual review to make sure they meet the standards and do not have data or metadata issues and inconsistencies. This status is selected by default when visiting all search views, including the Matrix page (refer to Basic Protocol, step 2).
Revoked	An error was found with the experiment after it became publicly available, so the status was changed to “revoked” to indicate that caution should be exercised before using the data. Some examples of errors are: 1. The data was not compliant with the ENCODE quality requirements 2. Issue discovered with experimental elements (antibody, biosample, etc.)
Archived	The dataset was superseded by another dataset that has higher quality, was collected and/or processed with newer technology, etc. The ENCODE DCC encourages use of the superseding experiment instead of the archived one.

a Additional information is available at https://www.encodeproject.org/help/getting‐started/status‐terms/.

### Filter search results using facets

**Figure 4:**
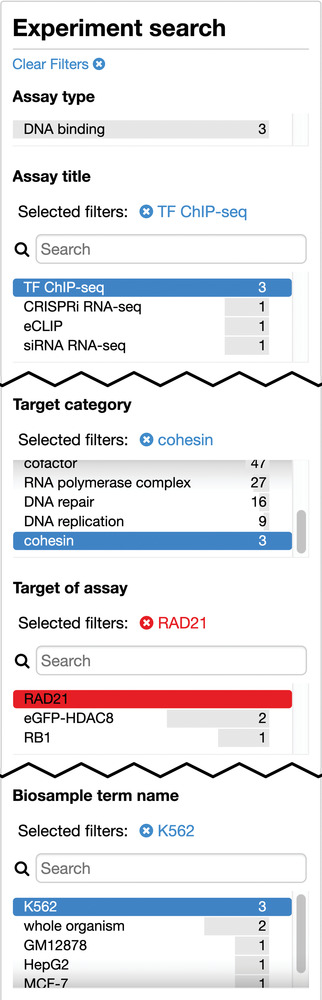
A truncated view of the facets with the items that should be selected after step 13 of the Basic Protocol.

5 The sidebar on the left side of the search page is populated with facets, which allow users to filter search results by different properties. Locate the “Assay title” facet and observe that the facet term “TF ChIP‐seq” is highlighted in blue, indicating that the search results have been filtered for experiments with an assay title of TF ChIP‐seq.
  - Clicking a cell in the “TF ChIP‐seq” column on the Experiment matrix page (see step 4) automatically selects the “TF ChIP‐seq” facet term.
  - In general, selecting a facet term applies that term as a filter, and automatically updates the displayed search results.
6 Scroll further down on the page and locate the “Biosample term name” facet. Observe that “K562” is already selected, indicating that the search results have been filtered for experiments with a biosample term name of K562.
  - Clicking a cell in the “K562” row on the Experiment matrix page (see step 4) automatically selects the “K562” facet term.
  - Selections can be made in more than one facet at a time. When such a selection is made, the combined filters possess an AND relationship. For example, the selection of TF ChIP‐seq in the “Assay title” facet and K562 in the “Biosample term name” facet returns only experiments that are TF ChIP‐seq assays AND use K562 cells as the biosample.
7 In the type‐ahead search box below the “Biosample term name” label, type DND‐41. The list of terms below the search box will be dynamically filtered.
  Because there are many biosample types to choose from, a type‐ahead search is available for this facet to help speed up the search process. This also applies to other facets with many terms, such as “Target of assay.”
8 Click “DND‐41” in the “Biosample term name” facet.
  - More than one facet term can be selected in a single facet simultaneously. Multiple selections in one facet represent an OR relationship between the selected facet terms. In this example, the selection of “K562” and “DND‐41” terms means the returned experiments may be on K562 or DND‐41 cells.
  - The URL for the current search is https://www.encodeproject.org/search/?type=Experiment&status=released&assay_title=TF+ChIP‐seq&biosample_ontology.term_name=K562&biosample_ontology.term_name=DND‐41.
9 Click “DND‐41” in the facet term list a second time to remove the filter for DND‐41 biosamples.
  - Users can also click the “DND‐41” link at the top of the facet after the words “Selected filters” to remove the filter. Both methods have the same result.
  - The URL for the current search is https://www.encodeproject.org/search/?type=Experiment&status=released&assay_title=TF+ChIP‐seq&biosample_ontology.term_name=K562.
10 Locate the “Target category” facet. Scroll through the list of terms and click on “cohesin.”
  - Targets have been categorized based on their Gene Ontology annotations in accordance with the methods described at https://www.encodeproject.org/target‐categorization/.
  - The URL for the current search is https://www.encodeproject.org/search/?type=Experiment&status=released&assay_title=TF+ChIP‐seq&biosample_ontology.term_name=K562&target.investigated_as=cohesin.
11 Locate the “Target of assay” facet. Hover the cursor over the “RAD21” facet term so that a red icon appears to the right. Click on the red icon to exclude experiments targeting RAD21.
  - The “Target of assay” most commonly applies to assays that utilize immunoprecipitation in their protocol, such as ChIP‐seq. In this context, the “target” refers to the DNA‐binding molecule targeted by the antibody for precipitation. However, the target property is sometimes also used for knockdown experiments or assays on genetically modified biosamples, such as shRNA RNA‐seq, in which case it refers to the gene target of the knockdown or modification.
  - The URL for the current search is https://www.encodeproject.org/search/?type=Experiment&status=released&assay_title=TF+ChIP‐seq&biosample_ontology.term_name=K562&target.investigated_as=cohesin&target.label%21=RAD21.
12 Scroll to the top of the page. At the top of the facet sidebar below the words “Experiment search,” click “Clear filters” to remove all selected filters.
13 Click the back button of the browser to undo the previous action. This will return to the previous query state for TF ChIP‐seq on K562 targeting cohesin‐related targets except for RAD21. The facet terms which should be selected at this stage are shown in Figure 4. As of September 2019, this search returned three experiments.

14 Review the list of search results, which displays the summaries of the experiments satisfying the selected filters.
  - Each result is labeled with a short title based on the assay performed and biosample used. Because this example search has filtered for both a specific assay and a biosample type, all the results are titled with “TF ChIP‐seq of K562.”
  - Below the short title there is a more descriptive biosample summary, followed by the target if applicable, the lab that performed the experiment, and the project the experiment belongs to, such as ENCODE or Roadmap.
  - Additional details about each experiment are located on the right side of each summary:
  - *Object type*: In this case, all results are Experiment objects. However, there are many object types modeled in the ENCODE database, which represent different experimental components. The data model is briefly discussed in the Commentary section of this article.
  - *The accession*: Each experiment on the ENCODE portal is given a unique and persistent identifier known as an accession, which can be used for citing ENCODE datasets. An example of an accession is ENCSR670JDQ. Users can directly access the record page for any accessioned object by appending its accession to https://www.encodeproject.org/.
  - *Status of the object*: Brief explanations of the different statuses are shown in Table 1, and further information is available at https://www.encodeproject.org/help/getting‐started/status‐terms/. Other object types aside from Experiments also have statuses.
  - *Audit flags*: The ENCODE DCC uses automated checks known as “audits” to flag objects in the database for potential data or metadata issues, some of which are outlined in Table 2. Once flagged, the DCC is able to work with production labs to address the issues and ensure that all data and metadata are up to quality standards. Audits are divided into different severity levels, represented as red, orange, or yellow icons, which serve as an indication to users of potential concerns to be aware of if they use the data in their own research. If flagged, the icon(s) will appear below the Status and can be clicked to show further details about the audits.
  - *Cart button*: This button allows users to add experiments to their cart. Further cart‐specific information is detailed in Support Protocol 2.

**Table 2:** Audit Flag Categories

Category	Description
Read coverage	Read depth or coverage issues for libraries. These standards were agreed upon by ENCODE production labs and are outlined in full on the data standards pages (https://www.encodeproject.org/data‐standards/).
Replication	Issues with replicate concordance or other replicate inconsistencies
Library complexity	Bottlenecking or library complexity issues, as outlined in the ENCODE Histone ChIP‐seq (https://www.encodeproject.org/chip‐seq/histone/) and Transcription Factor ChIP‐seq standards (https://www.encodeproject.org/chip‐seq/transcription_factor/)
Enrichment	Low SPOT scores for DNase‐seq experiments as outlined in the ENCODE DNase‐seq standards (https://www.encodeproject.org/data‐standards/dnase‐seq/)
Uniform pipeline requirements	Various pipeline issues, such as unexpected inconsistencies in read length, insufficient read length, and unknown platforms or other missing information
Antibody	Mismatches between antibody and target metadata or missing characterizations for antibodies
Metadata	Missing required metadata
Dataset consistency	Inconsistencies between different experiments grouped together in a series

^*a*^Additional information is available at https://www.encodeproject.org/data‐standards/audits/.

### Visit a single experiment page

**Figure 5:**
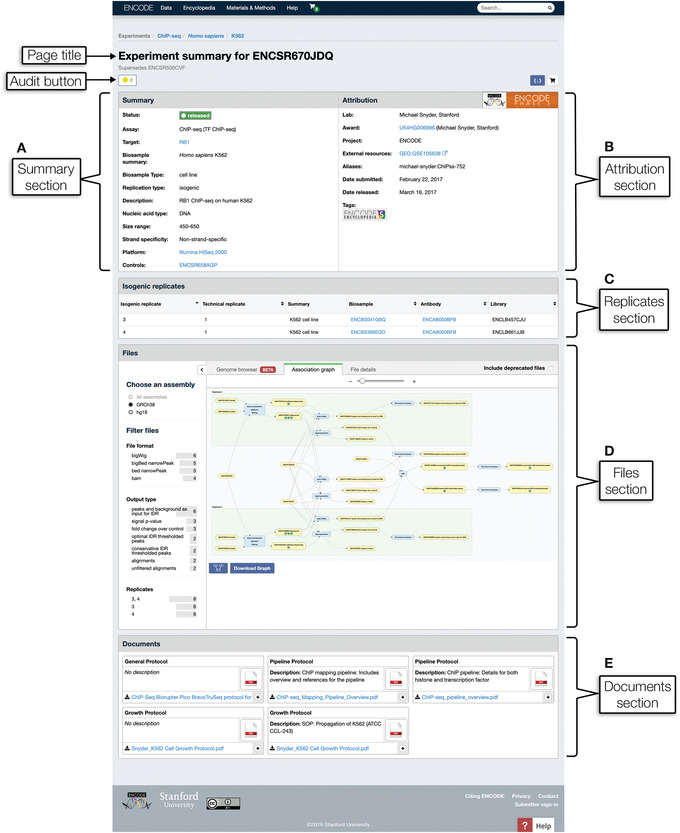
An experiment summary page. Below the page title and audits, the page is organized into distinct sections containing the following information: (**A**) Summary section: key info including but not limited to the assay performed, biosample used, assay target if applicable, platform, and controls. (**B**) Attribution section: information about the lab that performed the experiment and when the experiment was released. (**C**) Replicates section: table of experimental replicates with links to biosamples, antibodies, libraries, and genetic modifications when applicable. (**D**) Files section: information about the raw and processed data files generated from this experiment and subsequent analysis, provenance of data files as reflected in file association graph, and visualization of experiment‐specific genome tracks when applicable. (**E**) Documents section: links to additional protocol documents describing the experimental methods.

15 Click the short title corresponding to experiment ENCSR670JDQ to go to its experiment summary page, depicted in Figure 5. This experiment is also accessible at https://www.encodeproject.org/experiments/ENCSR670JDQ/.
  This page displays a more complete picture of the metadata, which is not shown in the summaries on the search result page, including links to related experimental components, data files, and documents.

16 Audit flags are also displayed on the experiment summary pages. Click the audit button, as labeled in Figure 5, to display a list of audits. For this experiment, the button appears as an unlabeled yellow icon with a number corresponding to the severity and number of the audit flags. The plus symbol to the left of each audit expands the audit further to display the audit flag details.
17 Scroll down the page to the Summary and Attribution sections.
  These sections contain general information about the experiment, such as the biosample, assay, and target, a link to a control experiment, and information about the lab that performed the experiment.
18 Scroll down the page to the Replicates section.
  This section contains information about the replicate(s) of the experiment and links to biosamples, genetic modifications, and antibodies used when applicable.
19 Scroll down the page to the Files section.
  - The Files section is divided into three tabs: Genome browser, Association graph, and File details. By default, the Files section displays the Association graph, which shows data provenance and derivation of downstream processed files. Use the slider above the graph to zoom in or out of the graph.
  - The Files details tab is discussed in step 22, and the Genome browser tab is described in more detail in Support Protocol 3.
20 Each node in the Association graph can be clicked to view more information about the node. Click on a yellow node, which represent files, to view a pop‐up containing the file's unique accession as well as other metadata such as the file size, output type, and submission date.
  Some file nodes may contain smaller, green circles representing quality control metrics associated with that file. These green circles are clickable and, like the file nodes, open a pop‐up with the quality metric (QC metric) values, plots, and other information if applicable. Click “Close” in the lower right‐hand corner of the pop‐ups when finished viewing the information.
21 Click on a blue node, which represents a step in the computational analysis pipeline, to view information about the software used, the inputs and outputs, and the general purpose of the relevant pipeline analysis step.
22 Click the “File details” tab to view a list of all files linked to this experiment.
  The files are presented in a table containing information about the file type, reference genome assembly for mapping, and submission date, with one file per row. A small download icon next to each file accession allows users to download a single file at a time.
23 Scroll further down to view the Documents section. Experiments may also have attached documents describing the experimental and/or computational protocols. Click the link for a particular document to download it.

## Support Protocol 1 BATCH DOWNLOADING

After identifying experiments of interest, users can download data associated with these experiments. Batch downloading provides a quick and simple way to download multiple files.

### Necessary Resources

#### Hardware

Computer with internet access

#### Software

Up‐to‐date web browser
Command‐line terminal
Command‐line UNIX utilities, available by default in macOS and Linux operating systems. For other systems, downloads for the utilities are available at:
  curl: https://curl.haxx.se/
  xargs: http://gnuwin32.sourceforge.net/packages/findutils.htm, https://www.gnu.org/software/findutils/findutils.html
A text editor
A spreadsheet application (optional)

1 Navigate to a search result page as described in the Basic Protocol, steps 1 to 14.
2 Click the “Download” button located above the list of results (Fig. 3). A pop‐up containing detailed instructions will appear.
3 In the lower right corner of the pop‐up, click the “Download” button to download a text file named files.txt containing a list of URLs of all files of each experiment returned in the search.
4 Open files.txt in a text editor.
5 The first line in the file is a metadata.tsv file download link. Enter this link into a web browser to download the file. Then, open metadata.tsv in a text editor or spreadsheet program.
  - Metadata.tsv is a table of metadata for each file listed in the files.txt file. This includes information about the file itself and its dataset, download links, and many other properties. The provided metadata fields are subject to occasional updates. The “File download URL” field contains HTTP links to the files, which are the same as those listed in the files.txt file. Another field labeled “s3_uri” contains Amazon Web Services (AWS) S3 Uniform Resource Identifiers (URIs) for each file, which can be used to access the files via cloud‐based tools. As of September 2019, these fields are located in columns 43 and 49, respectively.
  - Use a spreadsheet application or other tools to filter metadata.tsv by different properties for a subset of files of interest. For example, the grep UNIX command can be used to locate entries of files with the .bed.gz extension:
grep “.bed.gz” metadata.tsv
  Multiple commands can be piped together to extract only the “File download URL” field:
grep “.bed.gz” metadata.tsv | cut ‐f 43
6 The remaining lines in files.txt are download links for every file associated with the experiments returned in the initial search. There are multiple ways to download the files utilizing the provided links. One method is to use command‐line utilities such as curl to download every link in the text file. An example of such a command is:
xargs ‐L 1 curl ‐O ‐L < files.txt
  - Note that there may be many files to download. Before beginning to download, check that there is sufficient free disk space on the machine.
  - The method presented here is only one of many options. Many programming languages also have utilities or libraries that can be used to request HTTP links.
  - Users can also visit the links using a web browser to download files individually, similarly to the download of metadata.tsv in step 5.

## Support Protocol 2 USING THE CART TO DOWNLOAD FILES

The Cart feature allows users to save a set of experiments of interest and download files from these experiments later. Carts are saved per active browser session, so closing the browser tab or refreshing the tab erases items in the cart.

### Necessary Resources

#### Hardware

Computer with internet access

#### Software

Up‐to‐date web browser

**Figure 6:**
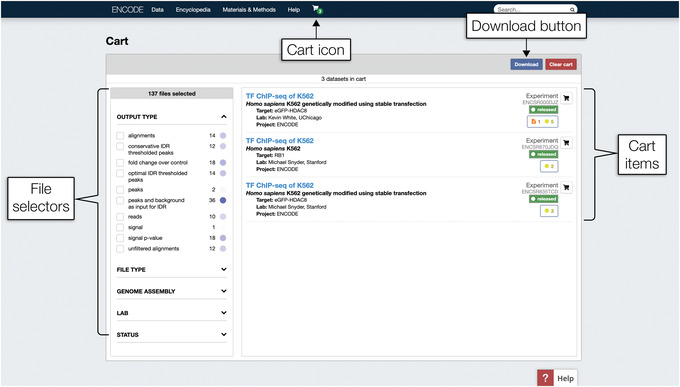
The Cart page. On the left are the file selectors. Although visually similar to the facet sidebar explored in the Basic Protocol, these file selectors only affect which files will be included in files.txt, introduced in Support Protocol 1. As selections are made, the number above the selectors, which reads “137 files selected” in this figure, will update dynamically. On the right is the list of experiments saved in the cart.

1 Navigate to a search result page as described in the Basic Protocol, steps 1 to 14.
2 Add a single experiment from a list of results to the cart by clicking the cart icon, which appears on the right side of each experiment summary.
  The cart icon is shaded in black when selected. The cart icon in the top toolbar displays the number of items in the cart.
3 Click the cart icon a second time to remove the experiment from the cart. The cart icon is no longer shaded in black after being deselected.
4 Click “Add all items to cart” in the upper‐right corner above the first search result to add every experiment in the list of search results to the cart (Fig. 3).
  Adding a large number of experiments could slow the browser down significantly. It is advised, though not mandatory, to have fewer than 500 experiments in the cart to avoid this issue.
5 Follow step 15 of the Basic Protocol to reach the individual experiment summary page for experiment ENCSR670JDQ. Click the cart icon located in the upper‐right corner of the page to remove it from the cart. Click the cart icon a second time to add the experiment back into the cart.
6 In the top toolbar, click the cart icon to open a drop‐down menu. In the menu, click “View cart” to visit the Cart page shown in Figure 6 and review the items in the cart. There should be three experiments listed on the right side of the page.
  If there are unwanted items in the cart, remove them by clicking the cart icons on the right side of the experiment summary.

7 The left side of the page contains the file selectors, which filter out the files included in the files.txt file. Locate the “Genome Assembly” file selector and click it to expand the options. Then, click the checkbox for “GRCh38”‐aligned files. The number of files above the file selector will automatically update. As of September 2019, it should read “60 files selected.”
  The file selectors are visually similar to the facet sidebar on the Search pages (Fig. 3), but these selectors only determine which files are included for download, and do not remove experiments from the cart.
8 Locate the “File type” file selector and expand it. Check the box for “bed narrowPeak.” The number of files is filtered to 15 bed narrowPeak files aligned to GRCh38.
  As with the Search page facets, users can make multiple selections in a file selector and selections in more than one file selector.
9 Click “Download” in the upper right above the list of cart items to open the same pop‐up seen in Support Protocol 1, step 2, and then download files.txt as in Support Protocol 1, step 3. The resulting files.txt file should contain only links to metadata.tsv and the 15 GRCh38‐aligned bed narrowPeak files of the experiments in the cart.
10 Continue following steps 4 to 6 of Support Protocol 1 to download the files.
  Once downloaded, users can proceed to analyzing the data as they wish. One example is using BEDTools to find overlapping peaks in two of the files (see Current Protocols article: Quinlan, 2014).

## Support Protocol 3 VISUALIZE DATA

The ENCODE portal supports visualization of analysis results such as signal or peak data from one or multiple experiments at once using Ensembl (Aken et al., 2017) or UCSC browsers (Kent et al., 2002). Visualization of supported HiC data is available through Juicebox browser (Durand et al., 2016). The ENCODE portal has also recently introduced embedded visualization of tracks directly on individual experiment pages using Valis genome browser (https://valis.bio/).

### Necessary Resources

#### Hardware

Computer with internet access

#### Software

Up‐to‐date web browser

### Visualize results from multiple experiments from a search page

1 To visualize files from multiple experiments at once, first navigate to a search result page according to the Basic Protocol, steps 1 to 14.
  Due to limitations of the genome browsers, there must be fewer than 100 results for the search in order to visualize them. If more than 100 results are returned, the “Visualize” button will be disabled and will instead read “Filter to 100 to visualize.”
2 Click the “Visualize” button at the top of the page. A pop‐up appears with different options grouped by the available reference genome assemblies.
3 In the “GRCh38” category, click “UCSC” to visualize the data from the experiments using the UCSC Genome Browser. Close the new window when done visualizing the tracks.

### Visualize results from a single experiment

**Figure 7:**
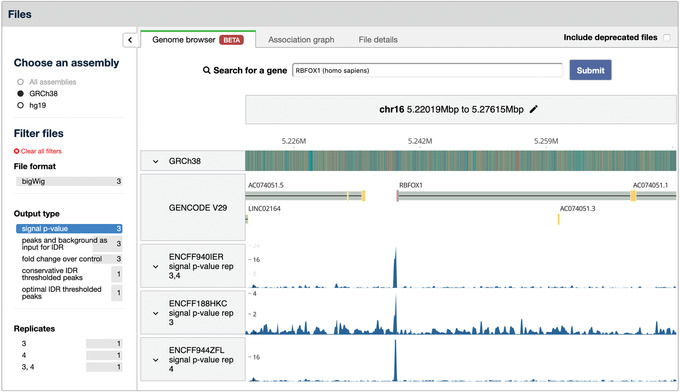
Genome browser tab in the Files section of an Experiment summary page. This tab contains the embedded Valis genome browser, which can be used to visualize signal and peaks tracks directly on the ENCODE portal. Filters to the left of the browser are used to select which tracks to visualize. Here, only the “signal *p*‐value” tracks are selected for visualization. The small arrow above the words “Choose an assembly” can be used to collapse and expand the filter sidebar.

4 To visualize results from a single experiment, navigate to an experiment summary page (Basic Protocol, step 15). Then, scroll down to the Files section.
5 Click on the “Genome browser” tab to visualize tracks using the embedded Valis genome browser.
  By default, all visualizable tracks will be shown. If available for the given reference genome assembly, gene and/or genome tracks are also displayed.
6 While hovering the mouse cursor over the tracks, click and drag or scroll to the left or right to move along the genome. Scroll up or down to change the zoom level of the tracks.
7 The sidebar in the Files section contains filters to switch reference genome assemblies or select specific tracks. Below the “Output type” header, select “signal *p*‐value” to view three signal tracks. This produces the view shown in Figure 7.
  Click the left‐facing arrow near the top of the sidebar to expand or collapse it. Click “Clear all filters” to remove all selected filters.

8 A search box for genes, labeled “Search for a gene,” is located above the tracks. Enter RBFOX1 and select the option that comes up, then click “Submit.” The genome browser will automatically refresh and load the location of the requested gene.
  The chromosome and coordinates are displayed below the search box. To directly edit the values, click the pencil icon to the right of the coordinates.
9 The ENCODE portal also supports visualization of tracks using external browsers. Click on the File details tab of the Files section. In the upper right corner of the table are two drop‐down menus, one for the assembly and the other for the genome browser, which can be UCSC, Ensembl, or Juicebox depending on the file formats of the available files.
10 Click the “Visualize” button to the right of the two menus to open the external genome browser in a new window.

## QUERY BUILDING AND PROGRAMMATIC ACCESS

The ENCODE portal is a user‐friendly interface for querying the ENCODE database. Queries can also be sent directly to the database using the Representational State Transfer (REST) API, bypassing the portal's user interface. The REST API accepts URLs in a typical HTTP format and returns metadata records as JSON objects. Search methods described in the Basic Protocol also make use of the REST API, but are “wrapped” in the portal's user interface for users who prefer to interact visually.

Facets (see Basic Protocol, step 5) represent a subset of commonly used query parameters. However, any property of any object type can potentially be used as a query parameter. These properties and object types are listed comprehensively on the ENCODE portal at https://www.encodeproject.org/profiles/?.

### Necessary Resources

#### Hardware

Computer with internet access

#### Software

Up‐to‐date web browser
Command line terminal
Command line utilities:
  curl: https://curl.haxx.se/
A text editor

1 Open any text editor and write down the first part of a query, which begins with https://www.encodeproject.org/search/?.
  At the end of this protocol, the completed query will be entered into a web browser or a terminal command.
2 Add the parameter type=Biosample to the query. All parameters take the format property=value. This example uses the “type” property with a value of “Biosample.” All objects share the “type” property, which indicates what category the object belongs to. The full query is now: https://www.encodeproject.org/search/?type=Biosample.
  - This query would return a list of all biosample objects on the portal.
  - The list of all object types is available on the Schemas page, which can be accessed by clicking the Materials & Methods drop‐down menu in the top toolbar and then clicking Schemas.
3 Add another parameter to the query by first appending “&” to the end of the query. Then, add the life_stage=adult parameter to the end. Add another parameter, biosample_ontology.organ_slims=eye, to filter by organ. All parameters will take the same format as the first one. The full query is now: https://www.encodeproject.org/search/?type=Biosample&life_stage=adult&biosample_ontology.organ_slims=eye.
  - Different object types have different properties. From the Schemas page (see step 2), click on any of the objects to view a list of their properties.
  - Other syntax tips and special parameters, as listed in Table 3, exist which can also be utilized for querying.

**Table 3:** Syntax for Query Building

Syntax	Parameter example	Description
=	assay_title=TF ChIP‐seq	The equal symbol (=) connects a property to its value
&	assay_title=DNase‐seq&assembly=mm10	The ampersand (&) symbol joins multiple parameters together
!=	assembly!=hg19	!= represents “not equals” and acts as a negation. In the example shown, the parameter would filter for objects with reference genome assemblies which are not hg19. This is equivalent to clicking the red exclusion icon in step 11 of the Basic Protocol.
*	treatment=*	The wildcard (*) means the parameter can have any value
.	biosample_ontology.term_name=HepG2	The data model of the ENCODE portal allows for certain objects to be embedded in others. Objects are able to access the properties of the objects embedded in them. The period joins properties and sub‐properties to form the “path” to an embedded property, akin to how the forward slash (/) is used in file directory paths. In this example, the biosample_ontology object is embedded in experiment objects, and this parameter accesses the term_name property of biosample_ontology.
format	format=json	format is a special property. Appending this to the query returns the page as a raw JSON object. The default value is HTML.
frame	frame=embedded	frame is a special property indicating how the ENCODE database should return a requested object. Some examples of values include: Object: No objects are embedded Embedded: All objects are embedded Raw: Object links are in UUID (Universally Unique Identifier) format, rather than the default @id format

4 Request the query by entering the following command, which uses curl to fetch a URL, into the command line terminal: curl ‐H “Accept: application/json” https://www.encodeproject.org/search/?type=Biosample&life_stage=adult&biosample_ontology.organ_slims=eye
  - After entering the command, the record for the biosample search should print directly to the terminal window. Like all objects on the portal, this record is a JSON object. Examples of terminal outputs are available at https://app.swaggerhub.com/apis‐docs/encodeproject/api/basic_search#/.
  - Other command‐line utilities can also be used here. Some programming languages also have suitable libraries for this purpose.
5 Alternatively, enter the query URL into a web browser to display the output as a list resembling the search page shown in Basic Protocol, step 4.

## GUIDELINES FOR UNDERSTANDING RESULTS

After completion of the basic and support protocols, users should have found a list of datasets, explored the metadata records for those datasets, and downloaded a file or files from the datasets. As of September 2019, the example search performed in the Basic Protocol returned three results. Those three experiments were linked to a total of 137 raw and processed data files, 15 of which were GRCh38‐aligned bed narrowPeak files.

When searching for data, it is possible to encounter a “No results found” page indicating that there are no ENCODE data available matching the query. This could be due to an error in the query, such as a typo in search parameters or use of properties not available for a given object type. To resolve these errors, carefully review the query and check the object's schema page to make sure that any properties used have been entered correctly, especially for the Alternate Protocol. Schema pages for all objects are available by clicking the Materials & Methods menu in the top toolbar and then clicking the “Schemas” menu item. If no results are found and the query is correct, that means that the ENCODE portal does not have such data available. Note that the “No results found” page is distinct from the “Not found” or “Bad Request” pages, which are only displayed when trying to visit an invalid URL and are equivalent to the 404 HTTP error code.

## COMMENTARY

### Background Information

The design of the ENCODE portal and its metadata model, elaborated upon in Gabdank et al. (2018), Hong et al. (2016), and Sloan et al. (2016), was driven by the need of the ENCODE Consortium for a data repository to store and distribute diverse genomic datasets, addressing both the needs of the production laboratories’ data and those of the end users seeking to explore, download, and analyze ENCODE data.

For data submitters and the DCC, it was crucial to accurately model all experimental variables such as the library preparation, input biological material, and reagents used, and integrate information about all experiments into a common model that could adapt to different assays, while still maintaining enough specificity to distinguish similar experiments and capture the unique characteristics of each assay. The ability of the model to accommodate future assay types and experimental elements was also a key factor.

Meanwhile, from the perspective of end users, it was important to ensure that the portal provided all necessary information to reproduce experiments and analyses and to ascertain the comparability of different datasets in an organized and searchable fashion. The system needed to be accessible to novice scientists and those with minimal computational background, but also allow large‐scale, programmatic access.

To fulfill these requirements, the DCC developed a hybrid relational‐object data store system known as SnoVault, as described in Hitz et al. (2017). All experimental components stored in the database are modeled as JSON objects, which is a file format that stores information as key‐value pairs. The JSON‐SCHEMA feature of the system allows validation of the different object types by checking each JSON object against a template or “schema,” and new objects can be modeled on demand by creating new schemas. These templates specify dependencies between properties of a particular object type, required properties, and enumerated lists of valid property values where applicable. Based on the templates, the system will automatically raise an error if an object fails to meet the specification, thereby ensuring data integrity. The encodeD system also has a JSON‐LD feature using a special “linkTo” property that allows links between different objects which, combined, are used in the ENCODE data model to represent an experiment or experimental component. Multiple objects can share links to the same object; for example, multiple different experiments could all use the same antibody. JSON‐LD enables some degree of denormalization by permitting objects to be returned embedded with full copies of linked objects rather than the link alone, which in turn allows users with an understanding of the data model to traverse objects and search with a great deal of flexibility. The portal front end converts these JSON objects into HTML pages viewable in a web browser, as explored in the Basic Protocol. Moreover, as shown in the Alternate Protocol, users can still easily fetch all objects in the database as JSON objects, then sort and manipulate metadata as needed.

The Basic and Alternate Protocols presented in this article take advantage of the encodeD data model to query the database and return precise results based on metadata properties. Although free text search of the ENCODE database is provided so that users have a breadth of search options, especially for straightforward queries, these protocols are recommended for their specificity in finding datasets.

### Critical Parameters and Troubleshooting

When searching the ENCODE database, using invalid parameters will return empty results. If users encounter problems finding data, it is recommended to carefully review the object types modeled and the properties for these objects. For advanced querying, it is also helpful to look at the data model (https://www.encodeproject.org/help/data‐organization/) to learn which objects have embedded objects that can be used for querying. Not all properties are available as facets, so for some particularly specific queries it may be necessary to check the object profiles to find metadata properties that will lead to the most precise results, and then apply some or all of the techniques described in the Alternate Protocol.

Because the ENCODE portal is a continually evolving resource, some features presented in this article may be modified in the future. The DCC recommends visiting the documentation pages on the portal, which are updated periodically, for more up‐to‐date information and examples.

### Funding

National Human Genome Research Institute at the National Institutes of Health [U24HG009397]. Funding for open access charge: National Human Genome Research Institute at the United States National Institutes of Health [U24HG009397].
